# A New Decision Model Approach for Health Technology Assessment and a Case Study for Dialysis Alternatives in Turkey

**DOI:** 10.3390/ijerph17103608

**Published:** 2020-05-21

**Authors:** Necla Öztürk, Hakan Tozan, Özalp Vayvay

**Affiliations:** 1 Department of Engineering Management, Marmara University, 34083 Istanbul, Turkey; 2 Affiliation Industrial Engineering Department, Medipol University, 34083 Istanbul, Turkey; htozan@medipol.edu.tr; 3 Faculty of Business, Marmara University, 34083 Istanbul, Turkey; ozalp@marmara.edu.tr

**Keywords:** HTA, decision-making, MCDA, AHP, TOPSIS, VIKOR, goal programming, fuzzy

## Abstract

Background: This paper presents a generic Multi-Criteria Decision Analysis (MCDA) model for Health Technology Assessment (HTA) decision-making, which can be applied to a wide range of HTA studies, regardless of the healthcare technology type under consideration. Methods: The HTA Core Model^®^ of EUnetHTA was chosen as a basis for the development of the MCDA model because of its common acceptance among European Union countries. Validation of MCDA4HTA was carried out by an application with the HTA study group of the Turkish Ministry of Health. The commitment of the decision-making group is completed via an online application of 10 different questionnaires. The Analytic Hierarchy Process (AHP) is used to determine the weights. Scores of the criteria in MCDA4HTA are gathered directly from the HTA report. The performance matrix in this application is run with fuzzy Technique for Order Preference by Similarity to Ideal Solution (TOPSIS), fuzzy Vise Kriterijumska Optimizacija I Kompromisno Resenje (VIKOR), and goal programming MCDA techniques. Results: Results for fuzzy VIKOR, fuzzy TOPSIS, and goal programming are 0.018, 0.309, and 0.191 for peritoneal dialysis and 0.978, 0.677, and 0.327 for hemodialysis, respectively. Conclusions: Peritoneal dialysis is found to be the best choice under the given circumstances, despite its higher costs to society. As an integrated decision-making model for HTA, MCDA4HTA supports both evidence-based decision policy and the transparent commitment of multi-disciplinary stakeholders.

## 1. Introduction

Health Technology Assessment (HTA) has been expanding worldwide steadily and rapidly for the last four decades. The motivation behind this expansion is the high healthcare expenditures faced by healthcare systems, the emergence of new health technologies, and the need to rationalize them in response to budget constraints [1,2]. Managers and health policy-makers can use evidence-based scientific information produced by researchers to direct the decision-making process. Thus, initiating new research is essential, which requires not only additional financing but also a considerable amount of time [3,4]. Organizations that operate formal HTA programs have an explicit objective to carefully consider the full range of clinical and economic evidence to capture decisions of acceptance, modification, or rejection on a rational basis [5]. 

The decision-making process in HTA involves various stakeholders, such as physicians, pharmacists, pharmacologists, and health economists, making it a multi-disciplinary process [6,7]. In the current constitution, decisions are made in a deliberative process that has two main restrictions: a limitation in the number of domains to be evaluated and transparency in the preferences and the value judgments of the multi-disciplinary stakeholders [8,9]. The quality of the decisions made by the public and healthcare providers additionally depends on the comprehensiveness and consistency of the decision-making process of the HTA [10,11,12].

The HTA Core Model^®^ is the methodological framework that was developed by the EUnetHTA in order to jointly produce and share HTA information. The HTA Core Model^®^ is composed of nine domains, which are divided into more specific topics and further issues. The nine domains of the HTA Core Model^®^ are health problems and their current use of technology; description and technical characteristics of technology; safety; clinical effectiveness; costs and economic evaluation; ethical analysis; organizational aspects; social aspects; and legal aspects [13,14,15,16].

The aim of this paper is to develop a generic Multi-Criteria Decision Analysis (MCDA) model for HTA decision-making that can be applied to a wide range of HTA studies, regardless of the healthcare technology type under consideration. For the development of the MCDA model, the HTA Core Model^®^ of EUnetHTA was chosen as a basis because of its common acceptance among European Union (EU) countries. The integrated decision-making model for the HTA developed in this study, the so-called MCDA4HTA, supports evidence-based decision policy, includes all the domains of the HTA Core Model^®^ comprehensively, and ensures the transparent commitment of multi-disciplinary stakeholders. Additionally, this paper includes an application of the proposed model via the HTA of the study “Role of Peritoneal Dialysis in Renal Care” in Turkey. 

### 1.1. MCDA and Methods Used in Healthcare

MCDA, as an important branch of operations research, aims to design mathematical and computational tools for selecting the best alternative among several choices by evaluating specific criteria. It has been widely applied in management, engineering, and environmental sciences [17,18,19,20,21,22,23,24,25,26,27]. 

**Figure 1:**
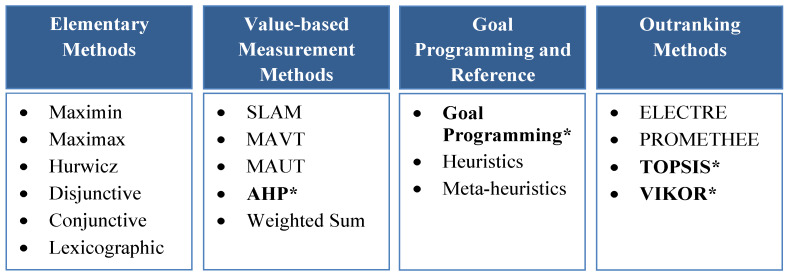
The Multi-Criteria Decision Analysis (MCDA) methods used in healthcare decision-making are elementary methods, value-based measurement methods, goal programming (GP) and reference methods, and outranking methods. The methods used in this paper are marked with “*”.

MCDA methods that are widely used in healthcare are grouped into four categories based on their level of complexity and the analytic model: elementary methods, value-based measurement methods, goal programming (GP) and reference methods, and outranking methods (Figure 1) [8,28,29,30]. 

Elementary methods are basic MCDA methods that require partial preference stimulation in the decision-making process [8]. Value-based measurement methods use quantitative measurements to define the fulfillment of the criteria and the priorities of the criteria to achieve the goal [29,30]. GP and reference methods are used in choice problems. In GP, the alternative or alternatives that are closest to achieving the pre-defined satisfactory levels of each criterion are derived. Outranking methods use pairwise comparisons for each criterion to define the preferences. The strength of evidence favoring the selection of one alternative is dependent on the preference information [8,28,31,32,33].

In this study’s application of the proposed model (MCDA4HTA), the Analytic Hierarchy Process (AHP), Technique for Order Preference by Similarity to Ideal Solution (TOPSIS), Vise Kriterijumska Optimizacija I Kompromisno Resenje (VIKOR), and goal programming MCDA methods are used. These methods are described in detail in the next sections. The purpose of using these MCDA methods is to enhance the reliability of the application by strengthening them with the advantages of different methods.

#### 1.1.1. The Analytic Hierarchy Process

AHP, first proposed by Thomas L. Saaty in the 1970s, is one of the MCDA methods that consider the relative importance of alternatives. AHP counts on both objective and subjective criteria and involves pairwise comparisons. AHP has become one of the most popular MCDA methods because of its flexibility in terms of its potential use for the analysis of complex problems and its user-friendliness [34]. AHP has been used in manufacturing, engineering, social science, and politics for selection, evaluation, resource allocation, and forecasting [35].

In any AHP model, the goal is set at the top of the hierarchical structure and is followed by the main criteria. If they exist, sub-criteria are written under the corresponding main criterion. The alternatives of the AHP model are set at the bottom of the hierarchy. 

The AHP methodology is based on the well-defined mathematical structure of consistent matrices and their associated right eigenvector’s ability to generate true or approximate weights. In AHP, the criteria or alternatives are compared with respect to a criterion in a natural, pairwise mode. Individual preferences are converted to ratio-scale weights, which can be combined into a linear additive weight for each alternative. The alternatives can then be compared and ranked.

Saaty suggested a scale for the definition of the relative importance of attributes that depends on the judgments of the decision-makers. The decision-makers define the relative importance of attributes by analyzing the criteria with respect to the goal or objective. The Saaty rating scale is used widely for pairwise comparisons [36,37].

#### 1.1.2. Technique for Order Preference by Similarity to Ideal Solution (TOPSIS)

Hwang and Yoon developed the Technique for Order Preference by Similarity to Ideal Solution (TOPSIS) method [38]. Here, the chosen alternative should have the shortest Euclidean distance from the ideal solution and the farthest from the negative ideal solution. In the ideal solution, which is a hypothetical solution, all criteria values correspond to the maximum criteria values in the database comprising the satisfying solutions. Conversely, the negative ideal solution is the hypothetical solution in which all criteria values correspond to the minimum criteria values in the database [32].

The solution generated by TOPSIS is the only solution that is both closest to the hypothetically best and the farthest from the hypothetically worst [39].

On the basis of their preference, decision-makers can decide on relative importance weights. The AHP method can be used to define the relative importance weights of criteria in a systematic way [32].

#### 1.1.3. Vise Kriterijumska Optimizacija I Kompromisno Resenje (VIKOR)

Više Kriterijumsko Kompromisno Rangiranje (VIKOR), meaning “Multi-criteria Optimization and Compromise Solution” in Serbian, is the compromise ranking method [40]. Yu and Zeleny first introduced a compromise solution to the literature [41,42]. It was later developed by Opricovic and Tzeng [43,44]. A compromise means an agreement founded by mutual concessions. The compromise solution is a feasible solution that is closest to the ideal solution [32]. VIKOR considers the weighted relation between the maximum group utility and minimum individual regret [44]. 

VIKOR is especially essential in situations in which the decision-maker is not able or does not know how to define preference at the beginning of system design. Compromise ranking can be performed by comparing the measure of closeness to the ideal alternative with the assumption of each alternative to be evaluated by each criterion. 

The main difference between TOPSIS and VIKOR methods was described by Opricovic and Tzeng: “the TOPSIS method determines a solution with the shortest distance from the ideal solution and the farthest distance from the negative ideal solution, but it does not consider the relative importance of these distances” [44].

#### 1.1.4. Goal Programming 

GP is an MCDA tool that was first mentioned by Charnes and Cooper [45]. GP, an extension of linear programming, is used as a multi-objective decision-making method. It can simultaneously and effectively cope with multiple independent or conflicting objectives. GP seeks to minimize an objective function, which can be defined as a combination of multi-dimensional absolute deviations from the target value [46].

Since GP permits non-homogeneous units of measure, it has wide usability and flexibility [47].

#### 1.1.5. Fuzzy Set Theory and Fuzzy Methods

In MCDA methods, the scores and weights of criteria are precise. However, in real life, with imprecision and uncertainty, it is not realistic to have precise assessments from experts or decision-makers [48,49]. It is both difficult and unrealistic to define an exact value for experts and decision-makers, so fuzzy and stochastic approaches are frequently used to describe and treat imprecise and uncertain elements [48]. To overcome the imprecision and uncertainty in expert and decision-maker preferences, fuzzy logic is introduced, and fuzzy versions of the Analytic Hierarchy Process (AHP), TOPSIS, and VIKOR are used in the application of the model provided by this study.

Fuzzy set theory was first introduced by L.A. Zadeh [49] in 1965, to deal with the vagueness of human thought. It was oriented toward the rationality of uncertainty due to imprecision or vagueness. The most important contribution of fuzzy set theory is its ability to represent vague data. Furthermore, the theory allows for a definition of mathematical operators and programming that can be applied to the fuzzy domain [49]. 

In fuzzy sets, instead of crisp values such as 0 and 1, the interval between these values is basically used to define the membership. Fuzzy sets were defined by Zadeh as a class of objects with a continuum of membership grades. The value is determined on the basis of a membership function [49].

### 1.2. MCDA Applications for HTA

MCDA takes multiple criteria simultaneously into account by using a set of qualitative and quantitative approaches [50]. In comparison with the deliberative process, MCDA is a more formal, structured, comprehensive, and transparent process [8].

Despite the great interest of national HTA agencies, such as the British National Institute for Health and Clinical Excellence (NICE) in the United Kingdom (UK), the German Institute for Quality and Efficiency in Health Care (IQWiG) in Germany, and the Canadian Agency for Drugs and Technologies in Health (CADTH) in Canada, the literature on the MCDA applications of HTA is limited. There are some publications on how to use MCDA in HTA. They describe in detail the requirements on MCDA applications and the MCDA methods that are frequently used in healthcare applications [8,29,31]. The literature containing MCDA applications in HTA studies is limited. 

In this study, two examples of MCDA applications of HTA are given: EVIDEM and Bariatric Surgery Selection [12,51]. The EVIDEM framework is the first MCDA application that was developed specifically for MCDA in healthcare or HTA studies. Additionally, it has been tested internationally in various applications in countries such as Canada, South Africa, Italy, and Spain in recent years [12,52,53,54,55,56]. The second example, Bariatric Surgery Selection for obesity treatment, is the first such application to HTA in Turkey [51]. Neither the EVIDEM framework nor Bariatric Surgery Selection has been used with the HTA Core Model^®^. 

#### 1.2.1. EVIDEM Framework

Goetghebeur and colleagues developed the evidence and value: impact on decision-making (EVIDEM) framework for healthcare decision-making to support the deliberative process, allow access to relevant evidence, and improve effective communication. The EVIDEM framework is a value matrix that includes different criteria and value-based measurement models used for MCDA [52].

The context of the decision in EVIDEM is structured in seven modules that cover the life cycle of a healthcare intervention. The framework is based on a value matrix, which is the quantification of the model. Here, the intrinsic value, together with the available evidence and quality of evidence, is assessed. Then, the extrinsic value is considered. The EVIDEM record, which is the result of the process, can be shared in a web-based collaborative database for transparency and application by other decision-makers. Evidence and decisions, updates, and the database can be accessed easily through the modular aspect [52].

The aim of developing the value matrix is to answer questions such as the value of a healthcare intervention with respect to its intrinsic characteristics. The value system for decision-makers and evidence-based evaluation defines the value estimate of an intervention as the combination of weights and scores [52].

#### 1.2.2. Bariatric Surgery Selection

One another application of MCDA in HTA was carried out by Karatas et al. in Turkey [51]. Karatas et al. developed a hybrid Visual C#-based decision support system (DSS) called the Decision-Making Tool Designed to Select (DEMATSEL), which includes MCDA methods such as AHP, fuzzy AHP, TOPSIS, fuzzy TOPSIS, VIKOR, fuzzy VIKOR, and goal programming [51]. The aim of developing DEMATSEL was to create a tool by which technological products and services could be assessed by their categorical values, and the best alternative could be chosen with a flexible and reliable process [51].

The model constructed by Karatas et al. to assess bariatric surgery in obesity treatment in Turkey through DEMATSEL is based on an available HTA report by the HTA Division of Ministry of Health (MoH) in Turkey. The alternative bariatric surgery treatments that are compared in the model are adjustable gastric banding, sleeve gastrectomy, and roux-en-Y gastric bypass. For the evaluation of these alternatives, five main criteria are defined: cost, risk, clinical characteristics, quality, and recovery from comorbidities [51].

HTA studies, as the source of comprehensive information or the basis for decision-making, need to be systematic, structured, transparent, comprehensive, consistent, flexible, bi-directional, and multi-disciplinary. The literature clearly shows that MCDA models are essential in HTA decision-making. The deliberative decision-making process in HTA should be supported by MCDA to impart the process with consistency and transparency. Researches in MCDA applications for traditional HTA decision-making and combating discussion on bridging HTA decision-making with MCDA process are limited in number but promise lots of improvements in the area [8].

## 2. Materials and Methods

### 2.1. Description of MCDA4HTA

In this paper, an MCDA model called MCDA4HTA is presented for use with the HTA Core Model^®^ of EUnetHTA. The motivation behind the development of MCDA4HTA is to provide an MCDA model that can be applied in various HTA studies, independent of the medical intervention or product. The first goal of the MCDA4HTA model is to develop an integrated decision-making model for HTA. Additionally, the HTA Core Model^®^ is currently used in many EU countries. A generic MCDA model with commonly defined criteria, which is integrated into the HTA Core Model^®^, could increase the use of MCDA in HTA. 

The criteria in the proposed model are defined from the HTA Core Model^®^. The MCDA4HTA model integrates two input sources: one is the HTA report based on the HTA Core Model^®^, and the other input is the judgment of decision-makers. The MCDA4HTA model can be applied to any Decision Support Software (DSS) that handles both linguistic terms and numerical data. 

#### 2.1.1. Structure of MCDA4HTA

**Figure 2:**
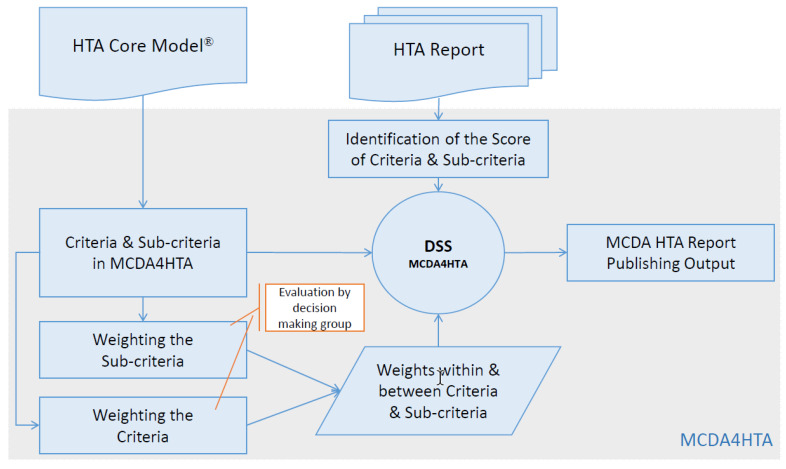
MCDA4HTA model: The criteria and sub-criteria are based on the Health Technology Assessment (HTA) Core Model^®^. The main inputs are the “weight” and the “score”. The importance among criteria and sub-criteria are defined by their weights, which are assigned by the decision-making group. The scores for criteria and sub-criteria are derived from the HTA Report under investigation.

The HTA Core Model^®^ for Medical and Surgical Interventions is the basis for the development of the MCDA4HTA model (Figure 2). Criteria and sub-criteria in MCDA4HTA are based on the HTA Core Model^®^. The nine domains of the HTA Core Model^®^ are accepted as the nine main criteria in MCDA4HTA. Sub-criteria under each main criterion are defined by critical evaluation of the underlying topics and issues in each domain. 

In MCDA4HTA, the main inputs are the “weight” and the “score”. The importance among criteria and sub-criteria are defined by their weights, which are assigned by the decision-making group. This group is usually formed by multi-disciplinary stakeholders such as physicians, pharmacists, pharmacologists, and health economists. The scores for criteria and sub-criteria in the proposed model are derived from the HTA Report under investigation. That means that the scores used in the MCDA4HTA decision-making model are based on evidence that is included in the HTA Report; they are not open to subjective judgments. After defining the scores and the weights for the criteria within the model, the next step is the execution of the performance matrix in the DSS chosen for the MCDA4HTA model. 

**Figure 3:**
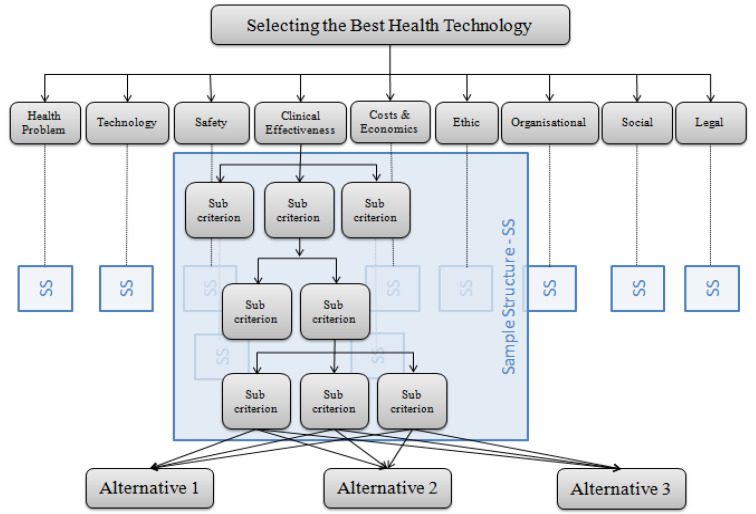
The hierarchical representation of the MCDA4HTA model. In the model, health problems, technology, safety, clinical effectiveness, costs and economics, ethical, organizational, social, and legal are nine first-level criteria. Because of the complexity, second-, third-, and fourth-level criteria are shown as in the sample structure. The hierarchies for each main criterion (first-level criteria) are given in Appendix A.

The hierarchical representation of AHP is used for the representation of the MCDA4HTA model (Figure 3 and Appendix A). The goal is set at the top of the hierarchical structure and is followed by the main criteria. If they exist, sub-criteria are written under the corresponding main criterion. The alternatives are set at the bottom of the hierarchy. The objective in the decision-making of HTA is usually to select the best technology, intervention, or drug alternative. The nine main criteria in MCDA4HTA (for ease of representation, short definitions are used in the schema) are given at the first level and are followed by the sub-criteria. The hierarchy under each main criterion is defined from the content of the topics and issues in a specific domain (Appendix A includes hierarchies for each main criterion). Additionally, an extensive analysis of the literature and detailed analysis of other MCDA models for HTA affect the sub-criteria defined from the HTA Core Model^®^. In defining the decision-making criteria, the priority is the fulfillment of the principles of MCDA modeling. 

One of the goals in developing MCDA4HTA is to generate a standard model that will be applicable in various decision-making processes of HTA. For that reason, after a preliminary definition of decision-making criteria through the process described above, another discussion round was performed with the support of HTA experts in MoH HTA Division in Turkey. 

In the MCDA4HTA decision-making model, there are nine main criteria covering 45 criteria in the second level, 115 criteria in the third level, and 113 criteria in the fourth level. The distribution of the number of criteria and sub-criteria can be seen in Table 1. 

#### 2.1.2. Defining the Weight of Criteria

The commitment of the decision-makers in the MCDA4HTA model is provided by the prioritization of the criteria that are under consideration. For prioritization, the scale suggested by Saaty is used [30,36,37]. The decision-makers define the relative importance of criteria by pairwise comparisons. In the MCDA4HTA decision-making model, the planned implementation of this phase is through a special type of questionnaire based on Saaty’s scale of relative importance. The MCDA4HTA decision-making model requires 10 different questionnaires for the definition of weights of the criteria. One of these questionnaires is for the prioritization of the nine main criteria among each other. The other nine questionnaires are used for each main criterion. The questionnaires consist of standard prioritization questions between pairwise comparisons of criteria based on Saaty’s rating scale. 

#### 2.1.3. Defining the Scores of Criteria

The scores are usually computed via the ratings of decision-makers with an MCDA method. Similar to weight determination, AHP or fuzzy AHP is widely used for the evaluation of the scores. However, the need to incorporate direct performance values for many of the MCDA problems has increased. Considering the concerns in the literature, the performance scores in the MCDA4HTA decision-making model are defined in such a way that they can be used as direct measures in an HTA report. In the model given in this study, the HTA report based on the HTA Core Model^®^ is proposed to be the source of the scores. The performance scores include both linguistic and numerical values. In this way, evidence-informed policy-making can be supported.

### 2.2. Decision Support Systems and DEMATSEL

Decision Support Systems (DSS) are software-based tools that are used by organizations to support and enhance their decision-making activities [57,58]. A DSS can provide a better perspective on the interactions of variables and the corresponding solution in complex situations that humans are unable to analyze [58]. There are numerous software programs developed as DSSs for generic use or specific problems and include either a single MCDA method or multiple MCDA methods, depending on the purpose of use. 

In the application of MCDA4HTA, the DEMATSEL DSS software is used. DEMATSEL is the hybrid DSS developed to integrate multiple MCDA methods and their fuzzy applications. DEMATSEL applies TOPSIS, fuzzy TOPSIS, VIKOR, fuzzy VIKOR, and oal programming to rank the alternatives. Additionally, it includes the determination of weights through AHP and fuzzy AHP methods. DEMATSEL supports both linguistic terms and numerical data. The user-friendly interface enables the direct input of data or the upload of data from exterior files [51].

### 2.3. Application of MCDA4HTA in HTA 

The MCDA4HTA model proposed is applied to “Role of Peritoneal Dialysis in Renal Care”, an HTA study performed by MoH Turkey [59]. This HTA study is based on the HTA Core Model^®^ of the EUnetHTA. For the application of MCDA4HTA in the Role of Peritoneal Dialysis in Renal Care HTA study, comprehensive online questionnaires are completed by the decision-making group, in order to define the weights of the criteria and sub-criteria designated from the HTA Core Model^®^. Secondly, scores for MCDA4HTA are determined directly from the Role of Peritoneal Dialysis in Renal Care HTA study.

The Saaty Rating Scale is used to collect the preferences of the participants via 10 different questionnaires applied online. The participants are asked to mention their preference among criteria/sub-criteria. For each questionnaire, online links are shared with the decision-making group of Dialysis HTA in MoH. All 10 questionnaires are shared over a six-month period. Full participation of the decision-making group, which consists of physicians, a dialysis physician, nephrologists, nurses, a hospital manager, a statistician, a healthcare economist, a pharmacist, and an industrial engineer for a size of 12 people, is agreed. The relative importance of criteria defined by weights is determined by solving a total of 85 pairwise matrices in AHP. The calculated weights for the application of MCDA4HTA to the Role of Peritoneal Dialysis in Renal Care HTA study are given in Appendix B. 

For the scores in the MCDA4HTA application of the Role of Peritoneal Dialysis in Renal Care, three different types of variables are used: numerical values, “yes” or “no” values, and linguistic terms. The numerical values are used as they are given in the report of MoH. For the interpretation of “yes” or “no”, the numerical values 1 and 0 are used, respectively. Linguistic variables are transformed via a 7-level linguistic term scale, which is given in Appendix C. The corresponding fuzzy numbers are assigned by DEMATSEL.

Furthermore, an example of online questionnaires is given in Appendix D.

## 3. Results

The performance matrix of the application of MCDA4HTA for the Role of Peritoneal Dialysis in Renal Care is provided in Appendix B, together with the objective function and crude weight for each criterion. Additionally, the normalized weights for the main criteria is presented in Appendix B. The analysis of MCDA4HTA is done with the DSS program DEMATSEL. DEMATSEL provides results by displaying the numerical values and a bar chart for the chosen MCDA technique. The DEMATSEL algorithm gives lower values to represent better solutions for VIKOR, TOPSIS, and goal programming.

Results for the Role of Peritoneal Dialysis in Renal Care with DEMATSEL (the performance matrix given in Appendix B) are in Table 2.

**Figure 4:**
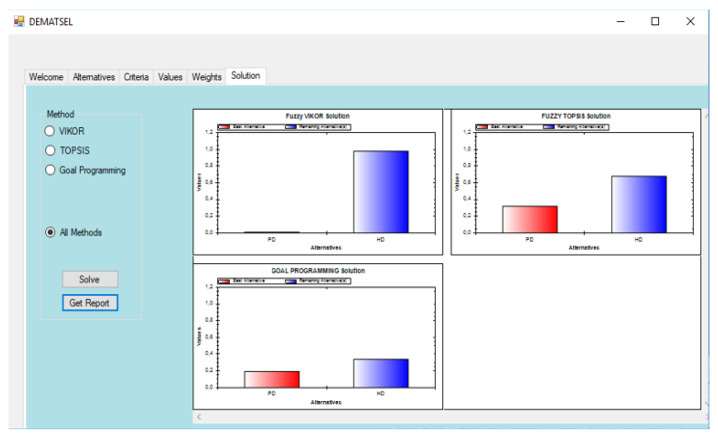
A screenshot of Decision-Making Tool Designed to Select (DEMATSEL) for results of the application of MCDA4HTA for the role of peritoneal dialysis in renal care.

Peritoneal dialysis (PD) has the lowest values for fuzzy VIKOR, fuzzy TOPSIS, and goal programming, i.e., 0.018, 0.309, and 0.191, respectively. The results are further depicted in Figure 4 with bar charts.

## 4. Discussion

Currently, the United Kingdom NICE, which makes recommendations to the National Health Service regarding new or existing healthcare technologies, uses the Incremental Cost-Effectiveness Ratio (ICER). ICER provides the incremental cost per quality-adjusted life years (QALY) gained by the patient per treatment. Although criteria such as severity and life-saving are also evaluated by ICERs, they have the limitation of ignoring important sources of value. Healthcare organizations in countries such as the Netherlands, France, and Belgium initiated criteria-based evaluations in their decision-making processes of priority setting or direct reimbursement evaluations [31]. MCDA is suggested for obtaining the value of QALY in many publications by transparent and consistent evaluations of multiple criteria explicitly [31].

MCDA models to support healthcare decisions have the potential to align the objectives of various healthcare decision-makers. Applications of MCDA models need to consider the way in which decision criteria and their weights are defined and the source of value evaluations. One of the major challenges of MCDA is gaining acceptance by all stakeholders [60].

Diaby and Goeree recommended generating case and pilot studies to show the value of MCDA with the cooperation of HTA authorities [8]. Although MCDA has various applications in many different fields, its development for HTA has been slow. This slow development can be explained by its development outside of the HTA field, but MCDA has origins in operations research. HTA authorities should provide an opportunity to bridge MCDA-based decision-making in HTA. Collaboration with MoH Turkey was possible for the application of the model in this paper.

The EVIDEM framework is one of the very first MCDA models in HTA decision-making. The framework is based on a value matrix, which is the quantification of the model. The performance of the criteria defined in the model is determined by the stakeholders via value judgments based on evidence. It is claimed that the decision-making in the deliberative process is supported through structured, segregated, and transparent access to evidence. It is suggested that a value estimate of the intervention for each stakeholder be calculated by a simple MCDA linear model. The advantage of EVIDEM is that it does not require complicated mathematical models or computation. Furthermore, it has been tested internationally by various applications, mainly in pharmaceutical evaluations in countries such as Canada, South Africa, Italy, and Spain [12,52,53,54,55,56].

One of the main differentiators between MCDA4HTA and other MCDA models in the literature, such as EVIDEM [52], bariatric surgery selection [51], and advance value tree [61], is the integrity of MCDA4HTA for HTA frameworks. In contrast to EVIDEM, MCDA4HTA incorporates direct values from HTA reports into the MCDA evaluation. Additionally, MCDA4HTA provides the evaluation of multiple objectives for the same criterion by using three different types of variables for scales: linguistic, numerical, and yes/no judgments. 

Involving various stakeholders transparently in the decision-making process has gained importance in recent years. In one recent study, patients were also included to express their preference for interventions [55]. MCDA4HTA proposes a very easy and intuitive way to incorporate the preferences of various stakeholders via online applications.

MCDA4HTA supports evidence-based decision policy by evaluating multiple criteria simultaneously and provides a transparent commitment of multi-disciplinary stakeholders. Furthermore, covering the whole HTA framework makes MCDA4HTA comprehensive. Flexible incorporation of the defined multiple criteria provides consistency.

MCDA4HTA was validated by an application in the Role of Peritoneal Dialysis in Renal Care. In this evaluation, PD was found to be the best apparent choice under the given circumstances, despite its higher costs to society.

One of the major limitations of this paper is the content of the HTA report. In the proposed MCDA4HTA, the weights of the criteria are defined by the multi-disciplinary decision-making group, while the scores of the criteria are retrieved from the HTA report. This obviously restricts the analysis by MCDA4HTA to the content of the HTA report. For that reason, the HTA report based on HTA Core Model^®^ should provide more numeric, objective, or quantifiable judgments. Including more evidence-based results will also improve the quality of the assessment by MCDA4HTA. Additionally, the analysis in the HTA report should cover as much as possible for all the comparators. Otherwise, any analysis done only for one technology alternative cannot be used in an MCDA evaluation.

## 5. Conclusions

Decision-making in healthcare is a complex process because it incorporates various aspects and involves different stakeholders from multiple functions. The increasing number of new health technologies and higher healthcare expenditures create high pressure on health authorities for rational, transparent, fair, and explicit healthcare decision-making processes. HTA promotes evidence-informed policy-making by providing evidence-based input to the decision-making process on the use of technology in healthcare. However, a rational, transparent, fair, and explicit healthcare decision-making process is essential. Various health authorities see MCDA as an aid to HTA-based decision-making. Although MCDA has wide use in other fields, its use in HTA-based decision-making is limited.

An integrated MCDA model, MCDA4HTA, is developed in this research for use in HTA decision-making. The current structure provided is specific for the HTA Core Model^®^ of the EUnetHTA. MCDA4HTA is the first model to be developed with the aim of integrating it into the HTA framework. It provides a transparent commitment of multi-disciplinary stakeholders and includes evidence within the scope of the HTA report. Moreover, MCDA4HTA provides a comprehensive and consistent evaluation of multiple criteria in order to make good decisions on behalf of the public.

MCDA4HTA is further validated by collaborating with MoH Turkey during their HTA study on the Role of Peritoneal Dialysis in Renal Care. The performance matrix in this application is run with the DSS program DEMATSEL, which includes TOPSIS, VIKOR, fuzzy TOPSIS, fuzzy VIKOR, and goal programming MCDA techniques. 

## Figures and Tables

**Table 1:** MCDA4HTA model criteria distribution table.

Main Criteria (1st Level)	2nd Level	3rd Level	4th Level
Health problem and current use of technology	4	8	14
Description and technical characteristics of technology	4	15	24
Safety	4	11	3
Clinical effectiveness	7	15	12
Cost and economic evaluation	6	6	-
Ethical analysis	5	15	15
Organizational aspects	5	14	13
Social aspects	3	10	24
Legal aspects	7	21	8

**Table 2:** Results for the application of MCDA4HTA for the Role of Peritoneal Dialysis in Renal Care.

Alternatives	Fuzzy Vikor	Fuzzy Topsis	Goal Programming
Peritoneal dialysis (PD)	0.018	0.309	0.191
Hemodialysis (HD)	0.978	0.677	0.327

**Figure A1:**
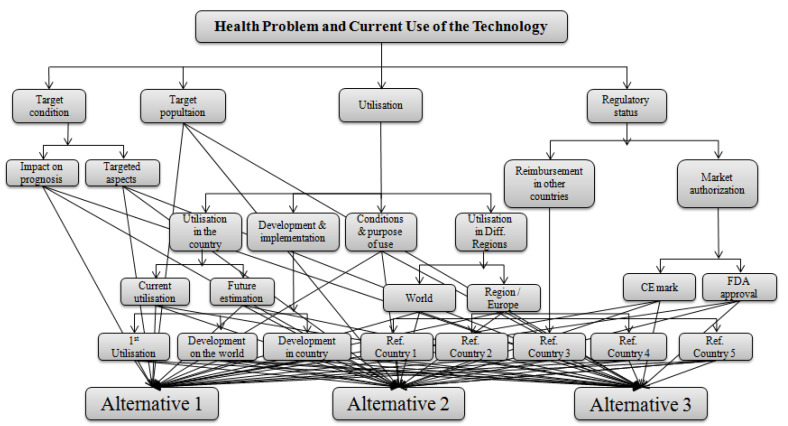
The hierarchical representation of health problem and current use of technology.

**Figure A2:**
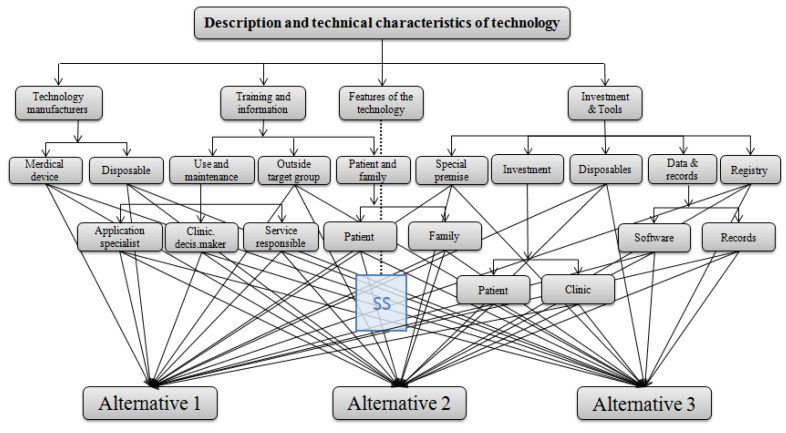
The hierarchical representation of description and technical characteristics of technology.

**Figure A3:**
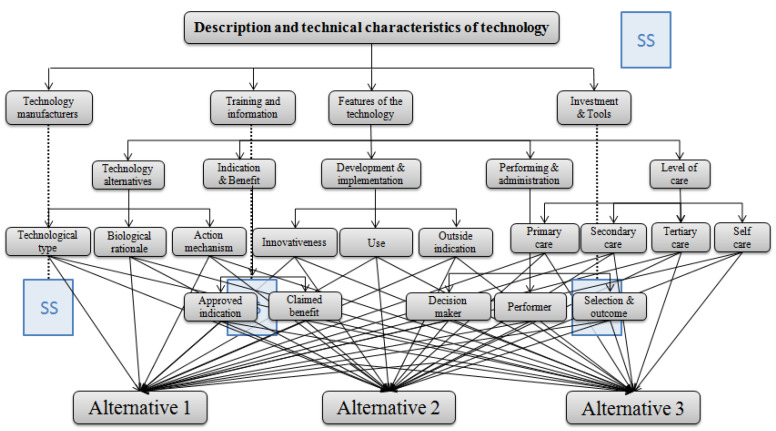
The hierarchical representation of description and technical characteristics of technology (cont.).

**Figure A4:**
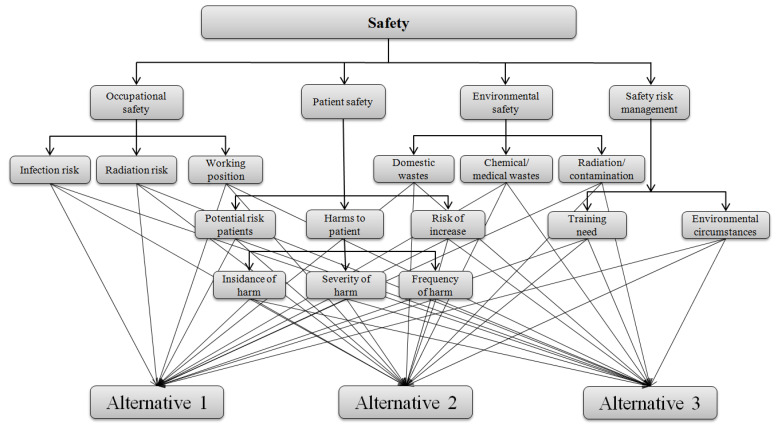
The hierarchical representation of safety.

**Figure A5:**
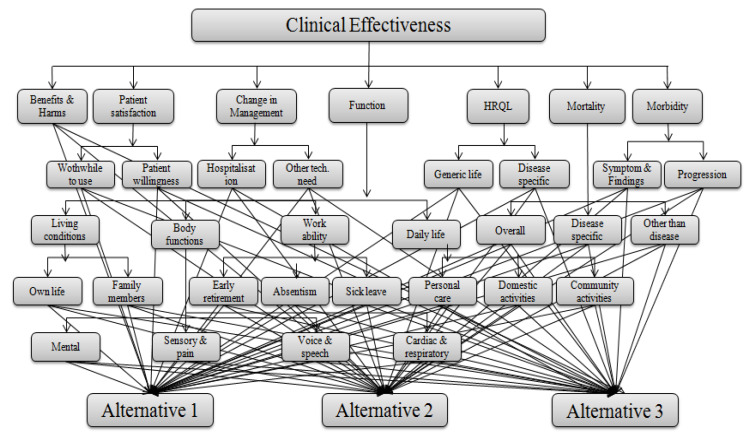
The hierarchical representation of clinical effectiveness.

**Figure A6:**
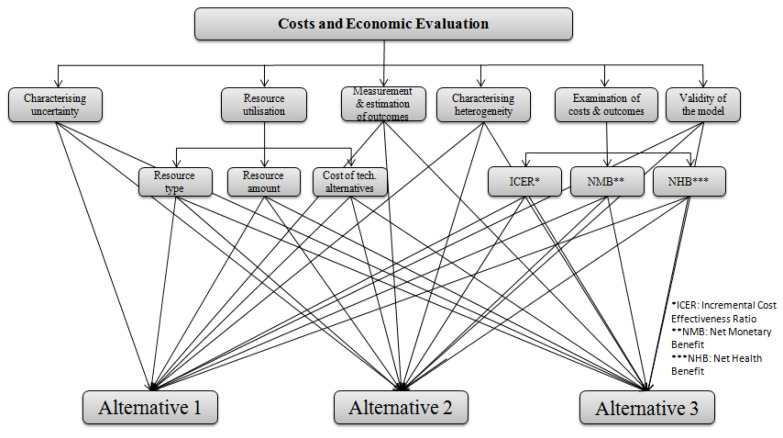
The hierarchical representation of costs and economic evaluation.

**Figure A7:**
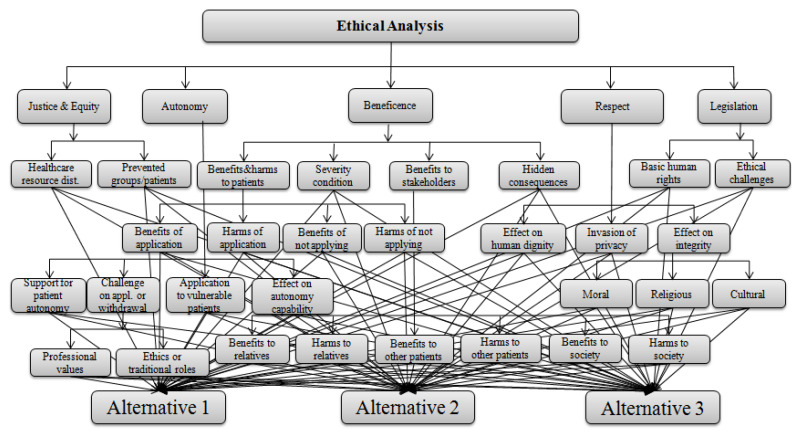
The hierarchical representation of ethical analysis.

**Figure A8:**
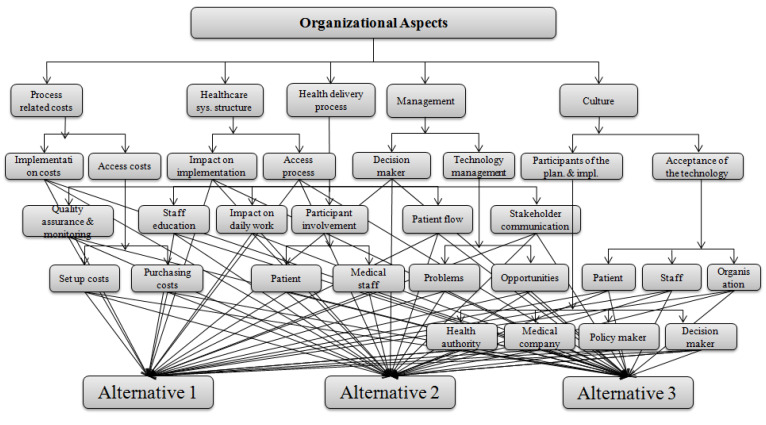
The hierarchical representation of organizational aspects.

**Figure A9:**
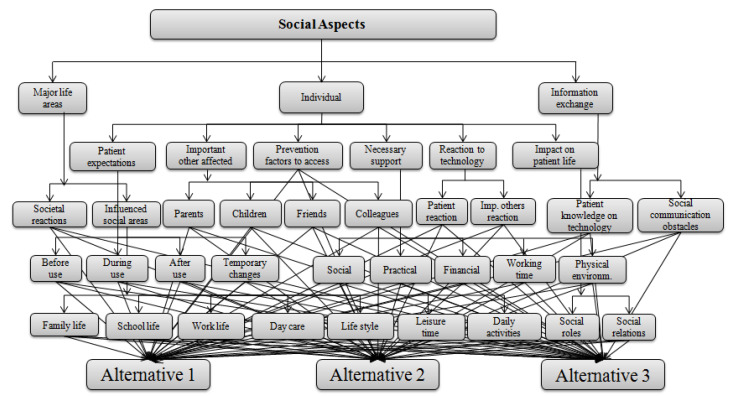
The hierarchical representation of social aspects.

**Figure A10:**
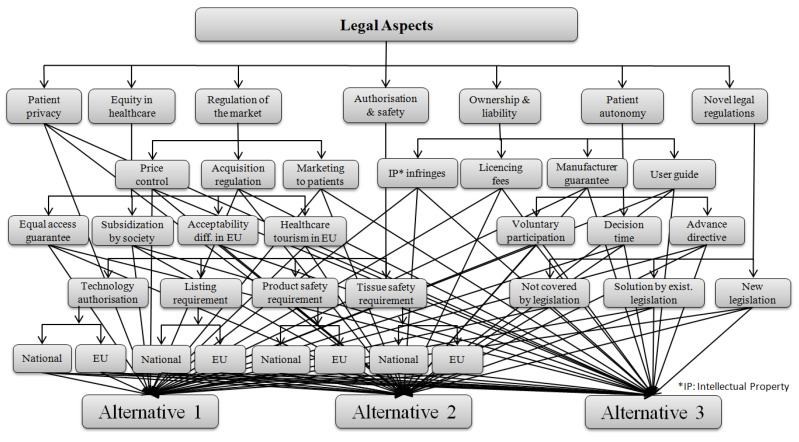
The hierarchical representation of legal aspects.

**Figure A11:**
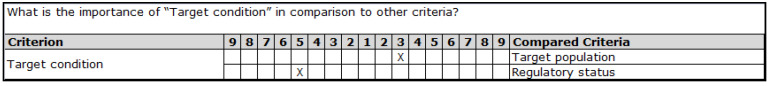
Selection of preference between criteria.

**Figure A12:**
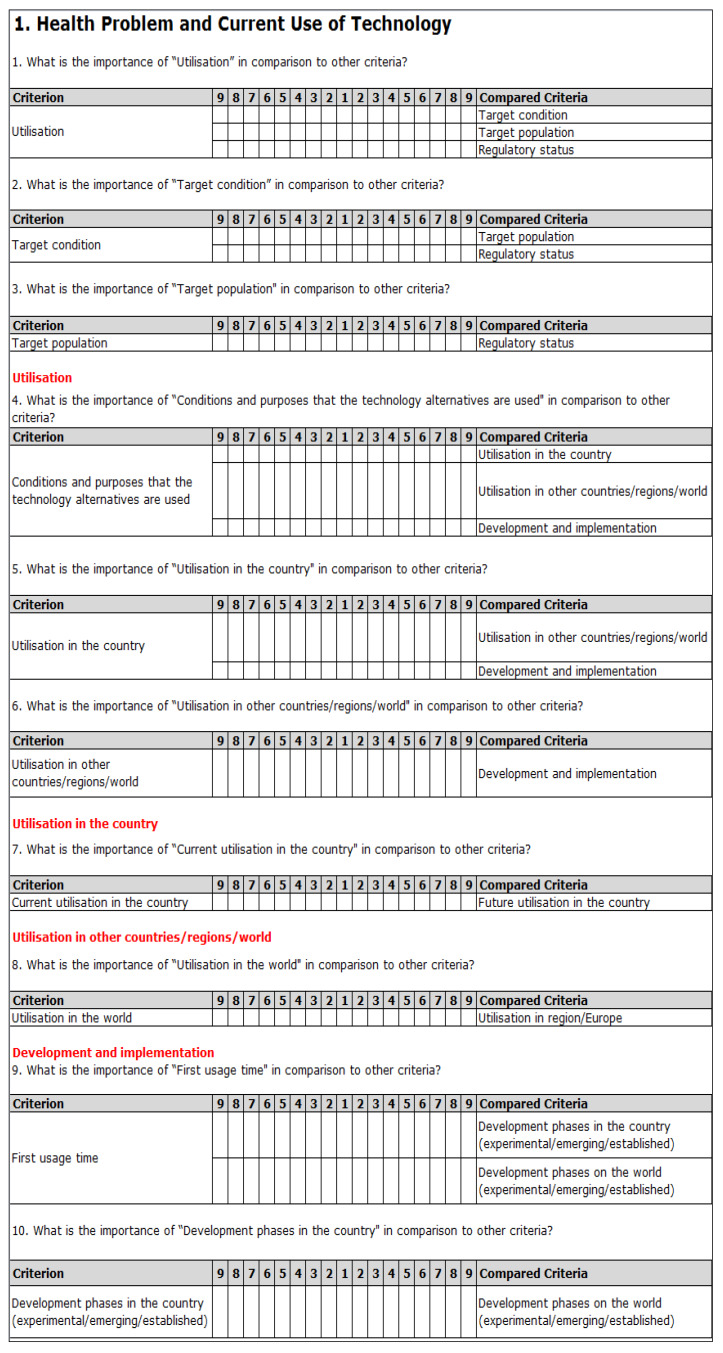
Questionnaire for health problem and current use of technology.

## Appendix A. Hierarchical Structure of the Main Criteria

### Appendix A.1. Health Problem and Current Use of Technology

Health problem and current use of technology criterion includes four sub-criteria in the second level, eight sub-criteria in the third level, and 14 sub-criteria in the fourth level.

### Appendix A.2. Description and Technical Characteristics of Technology

Description and technical characteristics of technology in MCDA4HTA model includes four sub-criteria in the second level, 15 sub-criteria in the third level, and 24 sub-criteria in the fourth level. Hierarchical structure under technology manufacturers, training and information, and investment and tools are given in the first graph (Figure A2), features of the technology with underlying sub-criteria is in the second graph (Figure A3).

### Appendix A.3. Safety

Safety in MCDA4HTA model includes four sub-criteria in the second level, 11 sub-criteria in the third level, and three sub-criteria in the fourth level.

### Appendix A.4. Clinical Effectiveness

Clinical in MCDA4HTA model effectiveness includes seven sub-criteria in the second level, 15 sub-criteria in the third level, and 12 sub-criteria in the fourth level.

### Appendix A.5. Costs and Economic Evaluation

Costs and economic evaluation in MCDA4HTA model includes six sub-criteria in the second level and six sub-criteria in the third level.

### Appendix A.6. Ethical Analysis

Ethical analysis in MCDA4HTA model includes five sub-criteria in the second level, 15 sub-criteria in the third level, and 15 sub-criteria in the fourth level.

### Appendix A.7. Organizational Aspects

Organizational aspects in MCDA4HTA model includes five sub-criteria in the second level, 14 sub-criteria in the third level, and 13 sub-criteria in the fourth level.

### Appendix A.8. Social Aspects

Social aspects in MCDA4HTA model includes three sub-criteria in the second level, 10 sub-criteria in the third level, and 24 sub-criteria in the fourth level.

### Appendix A.9. Legal Aspects

Legal Aspects in MCDA4HTA model includes seven sub-criteria in the second level, 21 sub-criteria in the third level and eight sub-criteria in the fourth level.

## Appendix B. Performance Matrix and Normalized Main Criteria Weights of MCDA4HTA for the Role of Peritoneal Dialysis in Renal Care HTA Study

Weights given in the table are the crude weights from for each third level or fourth level criteria. Some of the third level and fourth level criteria defined by careful evaluation of the HTA Core Model^®^ were not taken into consideration, because they were not considered in the HTA report of the Turkish Ministry of Health for the Role of Peritoneal Dialysis in Renal Care HTA. The normalized main criteria weights for the application of MCDA4HTA for the Role of Peritoneal Dialysis in Renal Care HTA Study are presented additionally after the performance matrix below.

**Table A1:** Performance matrix.

Criterion	Criterion Description	Objective Function	Peritoneal Dialysis (PD)	Hemodialysis (HD)	Weights
1.1.2	Utilisation in the country 2016	Maximize	2871	57052	0.359%
1.1.3.1	Utilisation in other countries/regions—World 2008	Maximize	196000	1764000	0.062%
1.1.3.2	Utilisation in other countries/regions—USA 2014	Maximize	9.,70%	90.30%	0.077%
1.2.1	Target condition—Impact of technology alternatives on the prognosis and course of condition (Life saving YES or NO)	Maximize	1	1	0.380%
1.3	Target population	Maximize	Very Good	Average	0.843%
1.4.1.1	Market authorization—CE Mark	Maximize	Bad	Good	0.458%
1.4.2	Reimbursement status	Maximize	Very Bad	Good	0.180%
1.4.2	Reimbursement status	Minimize	1225.00	537.24	0.180%
2.1.1	Technology manufacturers—Medical device manufacturers	Maximize	2.00	4.00	0.183%
2.1.2	Technology manufacturers—Disposable manufacturers	Maximize	2.00	15.00	0.183%
2.2.1.1	Training and information for Application specialists (nurse)	Minimize	3.00	6.00	0.153%
2.2.1.2	Training and information for Clinical decision maker (Nephrologists)	Minimize	3	3	0.379%
2.2.1.3	Training and information for Service and maintenance responsible (technician)	Maximize	Good	Bad	0.153%
2.2.2.1	Training and information for patient	Maximize	Bad	Good	0.304%
2.2.2.2	Training and information for his family	Maximize	1	0	0.090%
2.3.1.1	Technology alternatives—Technological type	Maximize	Best	Worst	0.017%
2.3.1.2	Technology alternatives—Biological rationale	Maximize	Best	Worst	0.019%
2.3.1.3	Technology alternatives—Action mechanism	Maximize	Bad	Good	0.019%
2.3.2.2	Claimed benefit	Maximize	Best	Average	0.128%
2.3.3.1	Innovativeness	Maximize	Average	Very Good	0.064%
2.3.3.2	Current use of technology	Maximize	1	1	0.047%
2.3.3.3	Use outside in current indication	Maximize	1	1	0.017%
2.3.4.1	Decision maker for starting and stopping the application	Maximize	Best	Very Bad	0.061%
2.3.4.2	User performing the technology	Maximize	Best	Very Bad	0.023%
2.3.4.3	User who selects the patients, interprets the outcome	Maximize	1	1	0.083%
2.3.5.1	Level of care—Self care	Maximize	Best	Very Bad	0.038%
2.3.5.3	Level of care—Secondary care	Minimize	0	1	0.023%
2.3.5.4	Level of care—Tertiary care	Minimize	0	1	0.023%
2.4.1.1	Required investment patients has to make	Maximize	Bad	Good	0.041%
2.4.1.2	Required investment clinics has to make	Maximize	Bad	Worst	0.124%
2.4.2	Required special premises	Maximize	1	1	0.337%
2.4.3	Required disposable—amount	Maximize	Average	Very Bad	0.084%
2.4.3	Required disposable—complexity	Maximize	Very Good	Bad	0.084%
2.4.4.1	Data and records essential to monitor the use—Software	Maximize	Bad	Good	0.054%
2.4.4.2	Data and records essential to monitor the use—Treatment records	Maximize	Best	Very Bad	0.054%
2.4.5	National registry	Maximize	2	2	0.093%
3.1.1	Harms technology can cause to the patient	Maximize	Very Bad	Bad	1.123%
3.2.1	Occupational safety—Infection risk for user	Minimize	0.03	0.0715	2.874%
3.2.2	Occupational safety—Radiation/contamination risk for user	Maximize	Good	Bad	0.890%
3.2.3	Working position	Maximize	Good	Bad	1.157%
3.3.1	Environmental safety—Domestic wastes	Maximize	Worst	Worst	0.467%
3.3.2	Environmental safety—Chemical/medical wastes	Maximize	Average	Worst	1.232%
3.3.3	Environmental safety—Radiation at the public level	Maximize	Average	Worst	2.053%
3.4.1	Safety risk management—Training need for users	Maximize	Average	Very Bad	2.260%
3.4.2	Safety risk management—Environmental circumstances for patients, citizens, and decision makers	Maximize	Average	Very Bad	1.180%
4.1.1.1	Effect of technology on body functions—Mental	Minimize	0.33	0.61	0.144%
4.1.1.4	Effect of technology on body functions—Cardiac & respiratory	Maximize	Bad	Worst	0.225%
4.1.1.5	Effect of technology on body functions—Gastrointestinal	Maximize	Worst	Bad	0.062%
4.1.1.6	Effect of technology on body functions—Skin functions	Maximize	Very Bad	Very Bad	0.059%
4.1.2	Effect of technology on work ability	Maximize	0.71	0.57	0.134%
4.1.3.1	Effect of technology on living conditions—own life	Maximize	Very Good	Bad	0.224%
4.1.3.2	Effect of technology on living conditions—Family members	Maximize	Worst	Very Bad	0.045%
4.1.4.2	Effect of technology on daily activities—Domestic activities	Maximize	Bad	Very Bad	0.051%
4.1.4.3	Effect of technology on daily activities—Community activities	Maximize	Bad	Very Bad	0.038%
4.4.1	Overall mortality—1st 3 years (HR)	Minimize	0.92	0.94	1.722%
4.4.1	Overall mortality—Mortality decrease rate (2015 vs. 1996)	Maximize	0.49	0.25	1.722%
4.5.2	Effect on progression—incident heart failure risk	Minimize	1.00	1.56	0.415%
4.5.2	Effect on progression—prevalent heart failure risk	Minimize	1.00	1.65	0.415%
4.5.2	Effect on progression—hip fractures	Minimize	1.00	1.60	0.415%
4.5.2	Effect on progression—ischemic stroke risk (in 10,000 patient years)	Minimize	100.10	61.60	0.415%
4.5.2	Effect on progression—hemorrhagic SVO (in 10,000 patient years)	Minimize	74.70	59.40	0.415%
4.5.2	Effect on progression—physician visit due to psychological problems	Minimize	0.08	0.09	0.415%
4.6.1	Hospitalization—all causes (in 1.25 years observation)	Minimize	0.85	0.75	0.331%
4.6.1	Hospitalization—infection (in 1.25 years observation)	Minimize	0.48	0.34	0.331%
4.6.1	Hospitalization in Australia over 1000 patients	Minimize	2.26	2.80	0.331%
4.6.1	Hospitalization time in Australia over 1000 patients	Minimize	13.30	10.30	0.331%
4.7.2	Patient satisfaction—Patient willingness on use of technology	Maximize	Good	Bad	1.641%
5.1.3	Cost of technological alternatives (annual cost/session of treatment)	Minimize	32098.81	26675.56	0.197%
5.1.3	Cost of technological alternatives (other direct costs excluding session costs)	Minimize	4015.26	4505.23	0.197%
5.1.3	Cost of technological alternatives (indirect costs)	Minimize	8825.42	7478.48	0.197%
5.2	Measurement and estimation of outcomes (QALYS)	Maximize	0.71	0.68	2.128%
5.3.1	Incremental cost effectiveness ratio – ICER – costs for 5 years	Minimize	185028.19	139226.60	0.285%
5.3.1	Incremental cost effectiveness ratio – ICER – effectiveness for 5 years	Maximize	2.89	2.36	0.285%
6.2.1	Autonomy—Application to vulnerable patients—infant & children	Maximize	Best	Very Bad	0.568%
6.2.1	Autonomy—Application to vulnerable patients—pregnant patients	Maximize	Best	Average	0.568%
6.2.2	Autonomy—Effect on patients capability or possibility to exercise autonomy	Maximize	Best	Good	0.494%
6.2.3	Autonomy—Need of additional information for patient autonomy	Maximize	Very Bad	Very Bad	0.769%
6.2.4.2	Challenge or change with application or withdrawal on ethics or traditional roles	Maximize	Worst	Very Bad	0.092%
6.3.1	Respect—Effects of technology on human dignity	Maximize	Worst	Worst	0.563%
6.3.2.1	Respect—Effects of technology on users moral integrity	Maximize	Worst	Worst	0.115%
6.3.2.2	Respect—Effects of technology on users religious integrity	Maximize	Good	Worst	0.018%
6.3.2.3	Respect—Effects of technology on users cultural integrity	Maximize	Good	Good	0.028%
6.3.3	Respect—Invasion of users privacy	Maximize	Good	Average	0.618%
6.4.1	Justice and equity—Effect of technology implementation or withdrawal on health care resource distribution	Maximize	Very Bad	Worst	1.482%
7.1.1	Health delivery process—Impact on daily work	Maximize	Bad	Worst	0.135%
7.1.2	Health delivery process—Patient/participant flow with the new technology	Maximize	Bad	Worst	0.122%
7.1.3.1	Health delivery process—Patient participation	Maximize	Best	Bad	0.106%
7.1.3.2	Health delivery process—Healthcare professional participation	Maximize	Average	Worst	0.038%
7.1.4	Health delivery process—Education and training for staff	Maximize	Worst	Average	0.639%
7.1.5	Health delivery process—Stakeholder co—operation and communication	Maximize	Bad	Bad	0.399%
7.2.1	Healthcare system structure—Impact on implementation in terms of centralization	Maximize	Average	Very Good	0.708%
7.2.2	Healthcare system structure—Process in ensuring access of patients and participants to the technology	Maximize	Worst	Very Good	1.415%
7.3.1.1	Purchasing process	Maximize	Average	Worst	0.384%
7.3.1.2	Set up process	Maximize	Average	Very Bad	0.313%
7.3.2	Budget impact of implementing the technology	Maximize	Very Bad	Very Bad	0.702%
7.4.1.1	Management—Problems	Maximize	Good	Bad	0.216%
7.4.1.2	Management—Opportunities	Maximize	Good	Bad	0.210%
7.4.2	Decision maker on eligibility of people for the use of technology	Maximize	1	1	0.709%
7.5.1.1	Acceptance of technology by organization	Maximize	Worst	Best	0.102%
7.5.1.2	Acceptance of technology by staff	Maximize	Bad	Best	0.042%
7.5.1.3	Acceptance of technology by patient	Maximize	Very Good	Bad	0.279%
7.5.2.1	Health authority participation	Maximize	Worst	Average	0.209%
7.5.2.2	Medical company participation	Maximize	Best	Good	0.093%
7.5.2.3	Policy maker participation	Maximize	Worst	Average	0.131%
7.5.2.4	Decision maker participation	Maximize	Worst	Best	0.336%
8.1.2.1	Parents affected by use of the technology	Maximize	Worst	Very Bad	0.070%
8.1.2.2	Children affected by use of the technology	Maximize	Very Bad	Bad	0.183%
8.1.2.3	Friends affected by use of the technology	Maximize	Bad	Bad	0.019%
8.1.2.4	People at work affected by use of the technology	Maximize	Very Bad	Worst	0.036%
8.1.3.1	Social support needed by patients	Minimize	Best	Best	0.094%
8.1.3.2	Practical support needed by patients	Minimize	Best	Very Good	0.065%
8.1.3.3	Financial support needed by patients	Minimize	Best	Good	0.353%
8.1.3.4	Working time support needed by patients	Minimize	Good	Best	0.302%
8.1.3.5	Physical environment support needed by patients	Minimize	Best	Average	0.268%
8.1.4.1	Change in social roles due to use of technology	Maximize	Very Bad	Very Bad	0.971%
8.1.4.2	Maintenance of social relationships	Maximize	Bad	Very Bad	1.111%
8.1.5.1	Patient action/reaction to use of technology	Maximize	Bad	Worst	0.678%
8.1.5.2	Important others action/reaction to use of technology	Minimize	1.00	4.50	0.103%
8.1.6	Factors preventing participation of a group or persons	Minimize	Average	Average	1.870%
8.2.1.1	Influence on family life by use of technology	Maximize	Bad	Worst	0.039%
8.2.1.2	Influence on school life by use of technology	Maximize	Very Bad	Worst	0.039%
8.2.1.3	Influence on work life by use of technology	Maximize	Very Bad	Worst	0.039%
8.2.1.4	Influence on day care by use of technology	Maximize	Worst	Very Bad	0.039%
8.2.1.5	Influence on life style by use of technology	Maximize	Worst	Worst	0.039%
8.2.1.6	Influence on daily activities by use of technology	Maximize	Worst	Very Bad	0.039%
8.2.1.7	Influence on leisure time by use of technology	Maximize	Worst	Very Bad	0.039%
8.2.2	Impact on overall social level by introduction of the technology	Maximize	Worst	Worst	0.429%
8.3.1	Knowledge and understanding of the technology in patients	Maximize	Very Bad	Worst	0.806%
8.3.2	Social obstacles or prospects in the communication about the technology	Maximize	Worst	Very Bad	0.240%
9.1.1	Authorization of the technology alternatives	Maximize	Best	Best	0.296%
9.1.2	Listing of technology in national or European Union registries	Maximize	Best	Best	0.214%
9.1.3	Product safety requirement fulfillment of technology	Maximize	Best	Best	0.425%
9.2.3	Content of manufacturers guarantee	Maximize	Best	Best	0.213%
9.2.4	Comprehensiveness of the technology user guide	Maximize	Very Good	Best	0.154%
9.4	Privacy of the patient	Maximize	Best	Bad	3.277%

**Table A2:** Normalized Main Criteria Weights.

Criteria	Main Criteria (1st Level)	Normalized Weight
1	Health problem and current use of technology	4.93%
2	Description and technical characteristics of technology	3.58%
3	Safety	18.86%
4	Clinical effectiveness	22.37%
5	Cost and economic evaluation	7.28%
6	Ethical analysis	11.76%
7	Organizational aspects	7.68%
8	Social aspects	10.26%
9	Legal aspects	13.27%
	Total	100%

## Appendix C. Scale Used to Interpret the Linguistic Terms for the Given Definitions in the HTA Report

**Table A3:** Scale used to interpret the linguistic terms.

Linguistic Terms		Worst	Very Bad	Bad	Average	Good	Very Good	Best
**Definition**	**Positive**	Worse Not interested Very negative effect Lowest	Very bad Low interest Negative effect	Bad Negative signs	Average Neutral	Good Positive signs Some tendency	Very good Some involvement Positive effect	Best Completely involved Excellent effect Very high
	**Negative**		More problems			Less problems More needs	Too much needs	At most needs

## Appendix D. An Example for MCDA4HTA Questionnaires

Dear participant,

Decision-making is as hard as it is important, especially if there are more than one criteria. In case of having more than one criteria it is named “Multiple Criteria Decision-Making” or “Multiple Criteria Decision-Analysis”

The Health Technology Assessment (HTA) is a process in which decision makers evaluate many criteria simultaneously and decision makers are involved from different disciplines. Today, HTAs must be systematic, planned, transparent, inclusive, consistent, flexible, reproducible and broadly involved to be accepted by society.

This questionnaire is being conducted for a paper, in which a multi-criteria decision-analysis model is developed for the HTA Core Model of EUnetHTA’s. The criteria used in the model are based on the EUnetHTA Core Model.

We kindly ask you to determine the importance of criteria (between criteria) when choosing the best healthcare technology by using the “Saaty Rating Scale”.

Only one box should be marked on each line. The marked “X” indicates the importance of the criterion (in the left column) in comparison to the compared criterion (the criteria in the right column). The marking at right side indicates the power of the criterion being questioned and the marking in the left side indicates the power of the criterion being compared (compared criterion).

When “target condition” compared to the “target population” in choosing the best healthcare technology the “target population” is found to be moderately important compared to the “target condition”. When “target condition” compared to the “regulatory status” in choosing the best healthcare technology the “target condition” is found to be much more important compared to the “regulatory status”.

Thanks for your contribution.
